# Gender Inequalities in Health and Their Effect on the Economic Prosperity Represented by the GDP of Selected Developed Countries—Empirical Study

**DOI:** 10.3390/ijerph17103555

**Published:** 2020-05-19

**Authors:** Robert Stefko, Beata Gavurova, Viera Ivankova, Martin Rigelsky

**Affiliations:** 1 Faculty of Management, University of Prešov in Prešov, Konštantínova 16, 080 01 Prešov, Slovakia; robert.stefko@unipo.sk (R.S.); viera.ivankova@smail.unipo.sk (V.I.); martin.rigelsky@smail.unipo.sk (M.R.); 2 Center for Applied Economic Research, Faculty of Management and Economics, Tomas Bata University in Zlín, Mostní 5139, 760 00 Zlín, Czech Republic

**Keywords:** health inequalities, life expectancy, mortality, avoidable mortality, diseases, prosperity of economy, gross domestic product, gender health inequalities, health differences, OECD

## Abstract

The objective is to evaluate the relations between gender health inequalities and economic prosperity in the Organisation for Economic Co-operation and Development (OECD) countries. The groups included health indicators in the specification of men, women and gender inequalities: life expectancy, causes of mortality and avoidable mortality. The variable determining the economic prosperity was represented by the Gross Domestic Product (GDP). The analytical processing included descriptive analysis, analysis of differences and analysis of relationships. The regression analysis was presented as the main output of the research. Most of the significant gender differences in health showed a more positive outcome for women. It is possible to identify a certain relation between gender health inequalities and economic prosperity. If there is some reduction in gender inequalities in health, the economic prosperity will increase. The reduction seems to be more effective on the part of men than women. The output of the cluster analysis showed the relations of indicators evaluating the inequalities and the prosperity. The countries such as Luxembourg, Norway or Switzerland showed very positive outputs, on the other hand, the countries with a potential for the improvement are Lithuania, Latvia or Estonia. Overall, the policies should focus on reducing the inequalities in avoidable mortality as well as reducing the frequent diseases in younger people.

## 1. Introduction

The purpose of an effective health system is not only to improve the health of the population [1], but also to achieve equality in health [2,3]. Each public policy (not only health policy) should be focused on the fulfillment of health potential of population without disadvantages and obstacles [4]. Marmot [5] concluded that the health status of the population is an important aspect of economic life and countries can be judged on the basis of health outcomes and a fair distribution of health care across the social spectrum. Nevertheless, there is considerable inequality in health around the world [6]. Gallardo-Albarrán [7] highlighted the fact that the 20th century is characterized by great health benefits that have improved the lives of the world’s population. On the other hand, it points to the unbalanced progress that has caused considerable health inequalities between countries. This is evidenced by the fact that differences in health indicators also occur between OECD countries [8]. Health inequalities can be identified not only between countries, but also within countries [9] and between groups of population with different socioeconomic, racial or gender status [10,11,12]. On this basis, the health inequalities can be considered as a serious issue [13], also highlighted by the World Health Organization (WHO) that included improving health for all and reducing health inequalities as a part of the strategic objectives of Health 2020 [14]. All these facts also underline the need for efforts to examine this issue from an economic point of view. The purpose of the present study is to clarify the relations between gender inequalities in health and the prosperity of economies represented by GDP. The main idea of the present research arose from the efforts to understand and examine the world issue of health inequality as a huge economic burden.

## 2. Theoretical Background

The fact that the health affects the economic life of countries is well known. McMichael et al. [15] stated that the health of the population should be one of the main criteria in the debate on sustainable development of countries. There is also an idea that a good health status of human capital plays an important role in terms of economic life [16] and is a representative element of countries [17]. This may be the main reason, why many studies dealt with the relationship between health and economic outcomes of countries [18,19,20,21]. The evidence showed that better health of population creates greater economic gains in the form of increased productivity and long-term income [22]. Additionally, the improvements in mortality reduction may bring potential economic savings reflected in saved years of population lives [23]. Concerning avoidable deaths of the population, the findings of a study by Alkire et al. [24] demonstrated that the unjustified nature of these deaths results in economic losses in the form of countries’ GDP decline. All these findings clearly indicate that health outcomes have an economic impact, but it is also appropriate to focus on the economic effect of health inequalities.

First of all, it is important to clarify what health inequalities mean and what factors cause this phenomenon. A health inequality is a difference in health or in the most significant impacts on health that could potentially be influenced by policies; it is a disparity in which disadvantaged groups (such as the poor, racial/ethnic minorities, women or other) generate worse health outcomes or face a more serious health risk than more advantaged groups [25]. These health inequalities represent unfair differences in the health status of the population and are determined by many social, socioeconomic and environmental factors that interact with each other. More and more emphasis is placed on social determinants that are the basis of health differences [9], the importance of social determinants is also underlined by international organizations such as the WHO and the OECD that consider the health inequalities to be a serious problem [26,27]. Wilkinson and Marmot [28] identified ten topics that are linked with social determinants: the social gradient, stress, early life, social exclusion, work, unemployment, social support, addiction, food and transport. On the other hand, Mackenbach et al. [29] focused on the socioeconomic status as a factor that determines the health inequalities and the findings revealed that education, income, health-related behavior and access to health care are significant factors. There are also environmental factors of health inequalities [30,31], including green space that was examined by Richardson and Mitchell [32], who dealt with gender differences in the relationship between urban green space and health indicators represented by cardiovascular disease mortality, respiratory disease mortality, self-reported limiting long-term illness and lung cancer mortality. Last but not least, the financing of health care systems can play an important role in achieving positive or less positive health outcomes [33], while Michalski et al. [34] recommended that non-profit organizations, including health facilities, should manage their funding with regard to efficiency in social and economic environment. In any case, funding in the health sector can be a significant factor that can contribute to improving the health status, achieving the health potential of the population, as well as reducing the health inequalities [35,36,37].

All of the above-mentioned factors can cause health differences around the world. Braveman [25] identified three basic components for measuring the health inequality: (i) an indicator of health or a modifiable determinant of health; (ii) an indicator of social position, i.e., a way of categorizing people into different groups and (iii) a method for comparing the health indicator (or health determinant) across the different social strata. Based on this, the present study includes the following selected components: (i) life expectancy, causes of mortality, avoidable mortality; (ii) gender status (males and females) and (iii) analysis of differences. The first two components are discussed in the next part of the literature review and findings.

The gender inequalities in health were examined in many studies [38,39,40,41] and the evidence revealed differences between women and men in several health indicators. Singh-Manoux et al. [42] found excess among men in mortality and some excess among women in morbidity. There are findings that women show poorer outcomes in mobility than men [43,44]. Wijnhoven et al. [45] argued that a female disadvantage in the musculoskeletal pain may be explained by sex differences in vulnerability to risk factors, i.e., overweight and older age were more associated with women, and pain catastrophizing was more common among men. With a focus on avoidable mortality, the gender inequality was confirmed in several studies [46,47]. The low rate of gender inequalities in avoidable mortality were confirmed by Westerling [48], but the findings of other studies definitely revealed that men have a higher risk of death due to avoidable causes [49,50]. In general, male mortality is significantly higher than female mortality [51]. This fact is evident not only in all-cause mortality, but also in terms of specific causes of mortality, namely COPD (chronic obstructive pulmonary disease), asthma, ACOS (asthma-COPD overlap syndrome) [52,53,54] and cancer [55,56]. On the other hand, men have a lower rate of cardiovascular mortality [57]. With regard to stroke, the research findings are debatable, women under 75 years have a lower risk of stroke compared to men [58], but older women lose this advantage and have a higher stroke mortality, while the highest risk of stroke occurs at an older age [59,60]. Le et al. [61] argued that efforts to reduce male mortality due to cancers, circulatory disease and respiratory diseases might decrease this gender gap in life expectancy. The authors also pointed out that the inequality in mortality in the 60–79 age category significantly contributes to the gender gap. Based on the results of another study, it can be concluded that life expectancy and healthy life expectancy are also shorter in men than in women, meaning that men are at greater risk of dying, they die earlier and live fewer disease- and disability-free years than women [62]. Similar evidence was found in the research that dealt with differences in life expectancy and self-rated health. The results of this study revealed that women live longer but report poorer health than men [63], this agrees with the findings of another study confirming that women report worse health but men’s mortality is higher [64]. The fact that women live longer was confirmed in several studies [61,65]. On the other hand, other studies showed that women lived longer lives, but had more disabilities than men [66,67], these disabilities are reflected in a lower quality of life related to health and well-being in older life [68]. At this point, it is necessary to refer to the findings by Oksuzyan et al. [69], who also revealed that there are significant differences between the health status and survival of women and men. This phenomenon is called the male–female health–survival paradox. Men are physically stronger and less disabled, but have significantly higher mortality in all age categories than women. This phenomenon occurs for several reasons, including the biological differences such as immune system reactions, genetic factors, hormones and disease patterns. Differences in behavior, such as risk taking and unwillingness to seek and comply medical treatment, can also be an important reason. Another aspect is that part of the difference may be due to methodological challenges such as selective non-participation, insufficient reporting of health problems and delayed seeking of treatment in men [69].

Based on the above-mentioned knowledge, it is possible to deepen the topic of health inequalities and focus on its association with economic prosperity. Health inequalities are well-examined, but their association with economic dimension has been investigated in few studies [70]. Several studies were conducted in a similar field of research but with a focus on health inequalities in different socioeconomic perspectives. Concerning the health inequalities between groups with different socioeconomic status, the findings of a study by Politzer et al. [12] confirmed a substantial economic impact of health inequalities in Israel, where the total cost of health inequalities was 0.7%-1.6% of GDP. These authors also highlighted the possible economic benefits of policies that will improve health outcomes of some groups of the population. From an economic point of view, the health inequalities were associated with high health care expenditure, social security costs and reduced labor productivity [71]. Mackenbach et al. [71] examined the inequalities in self-rated health and mortality in EU countries and confirmed that health inequalities are a huge economic burden in terms of the prosperity of economies. The inequality-related losses cause not only deaths, but also health care and social security costs or losses in GDP. On the other hand, the economic benefits of reducing health inequalities are reflected in economic savings [11,72]. Based on the above-mentioned, the health inequalities represent a huge economic burden and the efforts to close this gap have long-term potential benefits for the economy [73].

All the above-mentioned findings confirm two essential facts: the health inequalities affect many economic aspects of the country, including GDP; and there is a considerable gender inequality in health around the world. In this respect, it is considered necessary to examine the effect of gender inequalities in health on the economic prosperity represented by GDP. The purpose of this study is to fill this gap in scientific research and to highlight the link between gender inequalities in health and the economic prosperity represented by GDP. Simultaneously, the research offers a new perspective for policy makers, who focus on economic development and are not always aware of the important role of health and health inequalities in economic life. Last but not least, the study respects all recommendations of OECD and WHO that sensitively perceive the issue of health inequalities and urge the policy makers to address this serious issue [27]. This is despite the fact that research findings show that health inequalities are gradually decreasing [74]. Thus, it is important to point out the economic effect of this reduction and it can be discussed whether it is more effective to reduce inequalities on the part of women or men.

## 3. Materials and Methods

The primary aim of the research in this study was to evaluate the relations between gender health inequalities and the economic prosperity of OECD countries. This aim was met by a series of analytical procedures that were divided into three main parts. In the first part, a descriptive analysis, an analysis of differences in selected health indicators between males and females, and an analysis of relationships between selected health indicators and economic prosperity were applied. The aim of this first part was to point out the statistical characteristics of selected variables, the existence of gender differences (inequalities) in health and also the existence of a relationship between health indicators and the indicator of economic prosperity represented by GDP. For this purpose, the following two research questions were formulated: (RQ I-a) Is there a significant difference in selected health indicators between women and men? (RQ II-a) Is there a significant relation between economic prosperity and selected health indicators in terms of gender inequality? The aim of the second part was to use a regression analysis to evaluate the significance of the effect of female health indicators, male health indicators and gender inequalities in health on the economic prosperity represented by GDP. For this purpose, the following three research questions were formulated: (RQ I-b) Is there an effect of selected female health indicators on the economic prosperity of countries? (RQ II-b) Is there an effect of selected male health indicators on the economic prosperity of countries? (RQ III-b) Is there an effect of gender inequalities in selected health indicators on the economic prosperity of countries? The third part presents the outputs of a cluster analysis in which the visualizations determine and assess groups of countries based on the evaluation of the outcomes of gender inequalities in health and the economic prosperity of OECD countries. For this purpose, the following three research questions were formulated: (RQ I-c) Are there any homogeneous groups of OECD countries in the relations of indicators evaluating the gender inequalities in life expectancy and indicators evaluating the economic prosperity? (RQ II-c) Are there any homogeneous groups of OECD countries in the relations of indicators evaluating the gender inequalities in the causes of mortality and indicators evaluating the economic prosperity? (RQ III-c) Are there any homogeneous groups of OECD countries in the relations of indicators evaluating the gender inequalities in avoidable mortality and indicators evaluating the economic prosperity?

The analyses included data from the OECD database, while the Health and Productivity sub-databases were used for collection [8]. These databases are internationally recognized in providing relevant data on their member countries. Four categories of data were used in the analytical procedures. Three categories consisted of male and female health indicators such as a life expectancy (LE), the causes of mortality (CE) and an avoidable mortality (AM). The fourth category consisted of the indicator of economic prosperity represented by GDP in USD per capita (current PPPs). The life expectancy category included 4 age-specific variables separately expressed for women and men: life expectancy at birth—LE_1, life expectancy at age 40 years—LE_2, life expectancy at age 60 years—LE_3, life expectancy at age 65 years—LE_4 and life expectancy at age 80 years—LE_5. The value of these variables represents the average number of years that a person at that age can be expected to live, assuming that age-specific mortality levels remain constant [75]. The category of the causes of mortality included 13 specific variables reported in deaths per 100,000 population in standardized rates and separately expressed for women and men. This category was composed of variables that reflect age-standardized death rates per 100,000 population for chosen causes that were calculated by the OECD Secretariat, using the total OECD population for 2010 as the reference population [76]: certain infectious and parasitic diseases—CM_1, neoplasms—CM_2, blood diseases and blood forming organs—CM_3, endocrine nutritional and metabolic diseases—CM_4, mental and behavioral disorders—CM_5, diseases of the nervous system—CM_6, diseases of the circulatory system—CM_7, diseases of the respiratory system—CM_8, diseases of the digestive system—CM_9, skin and subcutaneous tissue diseases—CM_10, diseases of the musculoskeletal system and connective tissue—CM_11, diseases of the genitourinary system—CM_12 and certain conditions originating in the perinatal period—CM_13. The third health category of avoidable mortality consisted of two variables separately expressed for women and men, namely: preventable mortality—AM_1 and treatable mortality—AM_2. Both indicators refer to premature mortality under age 75. The preventable mortality is defined as causes of death that can be mainly avoided through effective public health and primary prevention interventions (i.e., before the onset of diseases/injuries, to reduce incidence). The treatable (or amenable) mortality expresses the causes of death that can be mainly avoided through timely and effective health care interventions, including secondary prevention such as screening, and treatment (i.e., after the onset of diseases, to reduce case-fatality) [77]. The economic prosperity was examined through GDP in USD per capita (current PPPs). All OECD countries were included in the analytical processes: Australia (AUS), Austria (AUT), Belgium (BEL), Canada (CAN), Czech Republic (CZE), Denmark (DNK), Estonia (EST), Finland (FIN), France (FRA), Germany (DEU), Greece (GRC), Hungary (HUN), Chile (CHL), Iceland (ISL), Ireland (IRL), Israel (ISR), Italy (ITA), Japan (JPN), Korea (KOR), Latvia (LVA), Lithuania (LTU), Luxembourg (LUX), Mexico (MEX), Netherlands (NDL), New Zealand (NZL), Norway (NOR), Poland (POL), Portugal (POR), Slovak Republic (SVK), Slovenia (SVN), Spain (ESP), Sweden (SWE), Switzerland (CHE), Turkey (TUR), United Kingdom (GBR) and United States (USA). The most recent data was from 2016 and the oldest since 2010, the time range was adapted to the large number of missing data. In some observations, the OECD reports limitations such as i—break (for LE and CM), ii—difference in methodology (for CM and AM—Turkey) or iii—provisional value (for LE); this was the number of observations that could not significantly affect the results.

In order to fulfill the main objective, the whole analytical process was decomposed into three sections. The content of the first part was a descriptive analysis showing the basic statistical characteristics of analyzed variables, i.e., central tendencies, variability and location. Subsequently, a difference analysis was used to examine the presence of differences in health indicators between women and men. For this purpose, the normality was tested by the Shapiro–Wilk normality test (SW). The Wilcoxon test (W) was also used as a non-parametric test of the difference of two independent samples. The content of the second part was a regression analysis, in which the health indicators in the specification of men, women and gender inequalities were used as independent variables. The gender differences were expressed in absolute values. The regression analysis was preceded by the selection of the most appropriate model based on testing of assumptions. The F test for individual and/or time effects was used to test the significance of the time series effects. The Bonferroni outlier test [78] was used to test the presence of significant outliers. The variance inflation factors (VIF) method was applied to consider multicollinearity. The Breusch–Pagan test was applied to test the homogeneity of variability of residuals (homoscedasticity). The content of the third part was an analysis of impact that was carried out at two levels. The first was a multiple regression, followed by a simple regression as the second, while the panel models, the fixed and random effect model, and the Arrelano and White 1 methods were used to estimate the coefficients in the case of significant heteroscedasticity. The content of the last part was a cluster analysis (agglomerative hierarchical clustering), in which the selected indicators of gender health inequalities and economic prosperity (represented by GDP) were used. In the first step, these data were adjusted by the median for all years (2010–2016). Subsequently, the data were standardized. The standardization of the data had an output from 0 to 1, where 0 was the most negative value and 1 was the most positive value of the evaluation. After the standardization, the data were adjusted by the mean in each specific group of indicators and four new variables were created: three indicators evaluating the gender health inequalities (LE_eval, CM_eval and AM_eval) and one indicator evaluating the economic prosperity (GDPpc_eval). The quasi-optimal number of clusters was estimated using the Silhouette method (for average silhouette width). Based on the highest value of the agglomerative coefficient, the Ward’s method was used to estimate the clusters themselves. Dendrogram and two-dimensional cluster charts were used to visualize the individual clusters. The analytical data were processed in programming language R v. 3.6.2 in R Studio (RStudio, Inc., Boston, MA, U.S.) and the libraries such as lmtest, car, sandwich, plm, cluster, fclust and ggplo2 were used.

## 4. Results

The following section of this research was devoted to the process of applying analyses leading to the fulfillment of the main objective and was divided into three main parts. The first part contained a descriptive analysis, an analysis of differences in selected health indicators between men and women, and an analysis of relationships that assessed the correlations between selected health indicators and economic prosperity represented by GDP. The second part contained a regression analysis determining the effect of health indicators on economic prosperity. The last part was devoted to a cluster analysis and an evaluation of individual countries in terms of analyzed indicators representing gender inequalities in health and economic prosperity.

### 4.1. Descriptive analysis

Table 1 shows the descriptive analysis results of selected health variables as well as the difference test output. Variables were divided into three groups—life expectancy, causes of mortality and avoidable mortality. Descriptive analysis outputs are shown for both men and women. 

The previous Table 1 shows the basic outputs of descriptive analysis of selected variables determining the health status of men and women. The bottom of the table shows the output of the difference test, which reveals the gender inequalities. The individual variables determining health status were divided into three groups. With a focus on life expectancy, women had higher average values in all LE indicators, i.e., women lived longer on average. The last row of the table confirmed the significance of these differences. Based on the Wilcoxon test outputs, all variables show a significant difference at the *p*-value of less than 0.001. In the second group, which includes the causes of mortality, only CM_5 and CM_10 showed no significant difference at the *p*-value of less than 0.05. The following interpretations of the outputs in Table 1 refer to variables in which there was a significant difference. In all but one case, the frequency of deaths due to specific causes was higher in men. Women had a higher mortality rate only in CM_11. The last group contained the health variables of avoidable mortality. As expected, the test outputs showed a significant difference confirmed by the *p*-value of less than 0.001. Both variables were more positive in women, i.e., the frequency of deaths among women due to AM_1 and AM_2 was significantly lower than among men. Based on the above-mentioned, it can be considered that women show more positive health outcomes than men.

The economic prosperity of the countries was represented by the GDP per head of population in USD current prices PPPs (GDPpc). During the analyzed period, this prosperity indicator in selected countries had a minimum value of 15,258 and a maximum of 107,775. Focusing on the outputs of central tendency, the average value was 38790, while the 95% confidence interval for the average had a lower bound of 36,908.64 and an upper bound of 40,671.35, and the median value was 37,144. In terms of variability, the economic prosperity had a value of standard deviation of 15,164.38 and a median absolute deviation of 13,627.75. With a focus on location, GDPpc was characterized by a skewness of 1.61 and a kurtosis of 4.33, suggesting some deviations from the normal statistical distribution. The following Table 2 provides the descriptive characteristics of gender inequalities in selected health indicators.

The following Table 3 offers a univariate view of the relations of selected health indicators and economic prosperity, as well as the assessment of these relations. In order to take this aspect into account, a correlation analysis, Spearman’s ρ, was used.

As can be seen in Table 3, there was a relationship of health indicators and economic prosperity in most analyzed cases. With a focus on gender inequalities in health, most cases had a significant negative coefficient, i.e., if the gender inequalities in health decrease, the prosperity of economies will increase. The male and female outputs could be interpreted in a similar way. At this point, it should be noted that the previous table did not offer the outputs of relation, thus it could not be confirmed that the changes in health indicators affect the economic prosperity. An overview of which health indicators and how these indicators affected the economic prosperity is given in the following part that is devoted to the application of regression analysis.

### 4.2. The Effect of Gender Health Inequalities on the Prosperity of Economies

The following part of the analyses was devoted to assessing the effect of selected health indicators on the prosperity of economies of OECD member countries. In the first part, the assumptions for the application of multiple linear regression and the selected optimal model were assessed. Subsequently, the specific analytical parts were divided into groups according to individual categories of health indicators. Each of these parts included an assessment of the effect of health indicators, separately in the specification of women, men as well as health inequalities between men and women.

The previous Table 4 was divided into three parts according to the logical structure of the subsequent steps of this analytical section. The first part of this section included models analyzing the effect of LE variables, the second part was focused on the effect of CM variables and the third part deals with the effect of AM variables. In the first step of assessing the assumptions, the multicollinearity was considered using the variance inflation factors (VIF) method. If the variables had a VIF value higher than 5, these variables were highly correlated and should not be included in the regression model. Therefore, the variables shown in the VIF row were excluded from the analyses. Taking into account the fact that the data for certain years and for certain countries had entered the analyses, it is necessary to consider the effects which leave time series or countries in the data, when choosing the appropriate model. For this purpose, the F test was applied that did not show a significant effect in individual years (test outputs are not shown), but the effects occurred in countries at a level of significance given by the *p*-value of less than 0.001. Therefore, a panel regression model was considered, and it was necessary to decide whether a fixed effect model or a random effect model would be more appropriate. This decision was supported by the Hausman test that provided (through the *p*-value supported by the χ2 value) information on preferences of fixed effect for regression models—Model 1 F, Model 2 M and Model 3 F. In other models, a random effect was shown to be more appropriate. The presence of significant heteroscedasticity, which was assessed using the Breusch–Pagan test, could seriously affect the estimation of the coefficients. The presence of significant heteroscedasticity was confirmed in all models. The last row in Table 4 shows the selection of the most appropriate method of regression model for the test of effects.

#### 4.2.1. Life Expectancy—LE

The first part of the assessment of the effect of selected health indicators analyses the effects of LE variables on GDPpc. The most important element in the research was the rate of effects in individual models; in terms of importance, the coefficient of determination (R2) was considered secondary. The first two models (Model 1 F and Model 1 M) show the R2 value at positive intervals, and the last model (Model 1 Ing) shows the R2 value at the limit of acceptability.

When assessing the outputs of the regression models in Table 5, the interpretation in the first step was focused on Model 1 F, i.e., on the effects of women’s LE indicators on GDPpc. Based on the outputs, it was evident that both LE variables were significant. The LE_1 variable had a positive coefficient, thus if the life expectancy at birth of women is increased, the economic prosperity represented by GDP is expected to increase. Conversely, if the average life expectancy of women at age 80 years (LE_5) decreases, the economic prosperity will increase. This is based on a logical idea that the vast majority of the population is no longer productive at a given age, at the same time this group of population is characterized by a higher social and health expenditure due to poor health. With a focus on men, only the LE_1 variable, with a positive coefficient, had a significant effect on economic prosperity represented by GDP. Thus, with increasing life expectancy at the birth of men, the economic prosperity will increase. When comparing the *p*-values of the male and female models in the LE_1 variable, it can be noted that the male coefficient was more significant. Thus, secondarily it could be assumed that men are more productive. The last part of Table 5 assessed the effect of gender inequalities in health on the economic prosperity represented by GDP and it was evident that the most significant variable was LE_1 that had a negative coefficient. Thus, if a gender inequality is decreased in LE_1, the economic prosperity is expected to increase.

#### 4.2.2. Causes of Mortality—CM

The following part assesses the effect of selected CM variables on GDPpc. In all cases, the coefficient of determination (R2) shows acceptable values.

The Model 2 F, shown in the first part of Table 6, demonstrated the significant effects of selected causes of female mortality, in particular for CM_2 and CM_5 to CM_9. The variables CM_2, CM_7 and CM_9 had a negative coefficient, thus reducing women’s mortality for these causes will result in increased economic prosperity represented by the GDP. The other three significant coefficients had positive values (CM_5, CM_6 and CM_8), i.e., these female mortality rates increased, the economic prosperity was also expected to increase. This effect was due to the specificity of the diseases that reduce or completely prevent the patient’s productivity and also entail high treatment costs. The effect with the highest rate of statistical significance on economic prosperity was found for the CM_5 variable. Focusing on the male regression model (Model 2 M), the significant effects occurred in five indicators, while the most significant effect was found for CM_2 and with a small difference for CM_5 and CM_7. The variables CM_2 and CM_7 had a negative coefficient, thus if a male mortality due to these diseases decreased, the economic prosperity was expected to increase. Conversely, as with women, if a male mortality due to diseases in the CM_5 group increased, the economic prosperity was also expected to increase. When assessing the effect of gender inequalities in health on the economic prosperity represented by GDP, only two variables, CM_2 and CM_7, showed the significant effects. Both variables had a negative coefficient, thus if a gender inequality in these indicators decreases, an increase of the economic prosperity is expected.

#### 4.2.3. Avoidable Mortality—AM

The last part of the assessment of the effect of selected health indicators analyses the effects of AM variables on GDPpc. The most important element in the research was the rate of effects in individual models; in terms of importance, the coefficient of determination (R2) was considered secondary. The first two models (Model 3 F and Model 3 M) show an acceptable value of R2, and the last model (Model 3 Ing) shows the R2 value at the limit of acceptability.

The outputs of Table 5 in the part devoted to the female model (Model 3 F) show that both health indicators (AM_1 and AM_2) had a significant effect on the economic prosperity represented by GDP. Both variables had a negative coefficient, suggesting that if these avoidable mortality rates of women decrease, an increase of the economic prosperity was expected. A preventable mortality (AM_1) shows a higher significance, this fact is logical and very easy to interpret from an economic point of view—a treatable mortality represents higher economic losses than preventable mortality. In a model that assesses selected indicators from the perspective of men, a reduction in preventable mortality (AM_1) of men predicted an increase in the economic prosperity represented by the GDP. The last part of Table 7 shows the output of the effect of gender inequalities in preventable mortality on the economic prosperity, this effect could be assessed as significant. Based on a negative coefficient, it could be concluded that if gender inequality is decreased in AM_1, the economic prosperity is expected to increase.

#### 4.2.4. Simple Regression Analysis—A Univariate Approach to the Relations of Health and Economic Prosperity

The following part was devoted to the application of univariate analysis of the effect of health indicators on the prosperity of economies represented by GDP. This purpose was supported by the regression analysis, while the most appropriate model was selected based on the results of the F test for individual and/or time effects, the Hausman test and the Breusch–Pagan test. These tests examined the significance of the effect of time series (years), which did not appear to be significant in any case, the significance of the effect of countries, which appeared to be significant in all cases, and the presence of significant heteroscedasticity, which occurred in most of the tested models. The individual outputs of these tests to support the models are not shown. The following Table 8 shows the regression analysis outputs.

The previous Table 8 was divided into three parts, while the first part (Female) shows the effect of health indicators on economic prosperity in the female specification, the second part (Male) shows the relations in the male specification and the third part (Gender Inequalities) deals with the relations in the specification of gender inequalities. With a focus on Gender Inequalities, most health indicators confirmed a significant effect on the prosperity of economies. Significant effect can be found in life expectancy at birth (LE_1) in the case of men, of women, as well as in the case of the health inequalities between men and women, thus if the gender inequality in LE_1 decreases, an increase of the economic prosperity is expected. Based on the *p*-value and the determination coefficient, it could be concluded that the reduction seems to be more effective on the part of men than women, in terms of the prosperity of economies. This is based on the fact that men are more productive than women, on the other hand, the longevity of men is shorter, by analogy it means that if the LE_1 of men increases, the gender inequality will decrease and the prosperity of economies will increase. Very similar conclusions could be considered in all cases of the life expectancy variables except LE_5 (life expectancy at age 80 years). It is possible to speak of a decline in the individual productivity after reaching a certain age (e.g., retirement age—65 years), which creates a certain part of the population that is inactive and also burdens the budget, which may be reflected in the economic prosperity. In general, the productivity of people over the age of 80 is very low. With increasing age (over 40 years), the productivity of people is declining, thus the policy efforts to reduce the inequalities would be most effective in the case of younger people, in terms of the prosperity of economies. The significance of the effects on the economic prosperity was not confirmed in CM_1 (certain infectious and parasitic diseases). This may be due to the fact that the incidence of these diseases is in the less productive groups of the population, and the course of the disease from identification to cure or death takes a relatively short time (e.g., sepsis), which does not overburden the budget and therefore does not reduce the economic prosperity. In addition, it is not a frequent group of diseases compared to other. The significance was confirmed in CM_2. This group represents the neoplasms that also occur in productive groups of the population, and the costs associated with these diseases were high, thus the effect on the economic prosperity of countries was evident. The reduction seemed to be more effective on the part of men than women, in terms of the prosperity of economies. Probably, the main reason for not confirming the significance of the CM_3 diagnosis group (blood diseases and blood forming organs) was the very low incidence of these diseases. As the treatment of some diseases (e.g., aplastic anemia) is relatively costly, this fact would result in a coefficient with a positive value. Nevertheless, the nature of these diseases, which allows patients to be economically active to some extent, contributes to balancing of these costs. The effect of gender inequalities was not significant even in the CM_4 diagnosis group (endocrine nutritional and metabolic diseases). In a gender specification the effect was confirmed in women, thus reducing the incidence of these diseases in women would have a positive effect on the economic prosperity. The mentioned finding may be explained by the nature of diseases in this group, which can be different in terms of cost of treatment, duration of treatment, as well as in terms of the incidence of the disease in different productive categories of the population. Significant effect on the economic prosperity appeared in the CM_5 diagnosis group (mental and behavioral disorders), these diseases occurred frequently in the productive population, but also in the pre-productive or post-productive population. At the same time, these diseases are associated with higher costs, which have an effect on the economic prosperity. The effect of gender inequalities in CM_6 (diseases of the nervous system) was not significant, the significant effects with a positive coefficient were identified in both men and women, explaining by the high cost of treatment. The significance of the effect of diseases in the CM_7 group (diseases of the circulatory system) was confirmed and it could be considered that the reduction seemed to be more effective on the part of men than women, in terms of the prosperity of economies. The significance of the effects in the CM_8 diagnosis group (diseases of the respiratory system) was not confirmed in women, therefore it is logical that in this diagnosis group more attention should be paid to the reduction of diseases of men, in terms of the economic prosperity. The effect of the CM_9 diagnosis group (diseases of the digestive system) seemed to be significant and it could be concluded that an increased attention in reducing the gender inequalities should be paid to men. Given the frequency of diseases in the CM_10 diagnostic group (skin and subcutaneous tissue diseases) and CM_11 diagnostic group (diseases of the musculoskeletal system and connective tissue), it was expected that the significance of the effects would be not confirmed. When looking to the CM_12 diagnostic group (diseases of the genitourinary system), it is possible to talk about a significant effect on the economic prosperity in both men and gender inequalities, on the other hand in the case of women, the effect was not confirmed, by analogy the reduction of gender inequalities seems to be more effective on the part of men than women, in terms of the prosperity of economies. In the CM_13 diagnostic group (certain conditions originating in the perinatal period), a significant effect on the economic prosperity was not found, it can be explained mainly by a low frequency of incidence of the diseases of this diagnostic group compared to other diagnostic groups. The effects of AM_1 (preventable mortality) and AM_2 (treatable mortality) seemed to be significant. These groups included mainly the diseases that affect the productive part of the population. Overall, the most significant effects could be found in the variables of LE_1, CM_2, CM_7, AM_1 and AM_2, all of which had a negative coefficient. Thus, if the gender inequality in health decreases, the economic prosperity will increase. It can be discussed whether it is more effective to reduce the inequalities on the part of women or men. Based on the additional calculations, it could be argued that the efforts to reduce the negative values of these health indicators seemed to be more effective on the part of men than women, in terms of the prosperity of economies.

### 4.3. Cluster Analysis

In this part of the research, the OECD countries were evaluated on the basis of selected indicators of gender inequalities in health and economic prosperity represented by GDP. For the purpose of cluster analysis—agglomerative hierarchical clustering, the data were adjusted and in the first step, the median value was computed between individual years. Subsequently, the individual variables were standardized. The standardization of the indicators had an output from 0 to 1, where 0 was the most negative value and 1 was the most positive value. Thus, it is an assessment of countries where an output closer to 1 means a more positive result. After standardization, the data were adjusted by the arithmetic mean in each individual group of indicators. After these steps, four new variables were created: three indicators evaluating the gender health inequalities (LE_eval, CM_eval, AM_eval) and one indicator evaluating the economic prosperity (GDPpc_eval).

**Figure 1:**
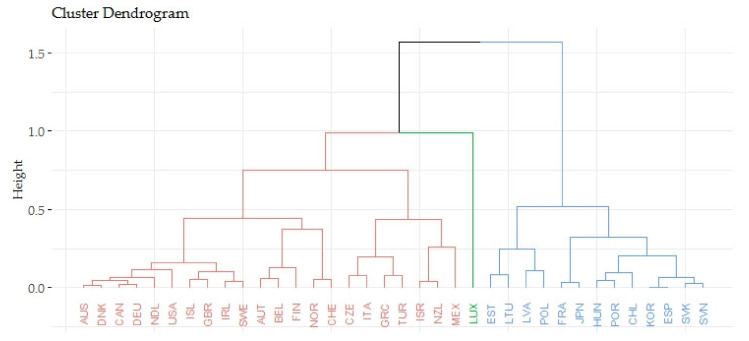
Hierarchical clusters of OECD countries—the relations of economic prosperity and gender inequalities in life expectancy.

Based on the agglomerative coefficient of approximately 0.9444, the Ward’s method was considered the most appropriate method. The Silhouette method recommended three clusters for the application. The following Figure 1 shows the output of the dendrogram, which determines the links of indicators evaluating the gender inequalities in life expectancy (LE_eval) and indicators evaluating the economic prosperity of countries (GDPpc_eval).

The previous Figure 1 focuses on the relations of indicators evaluating the gender inequalities in life expectancy (LE_eval) and indicators evaluating the prosperity of economies (GPPpc_eval). The countries were divided into three groups based on the smallest differences within the cluster and the largest differences between other countries (clusters). The first cluster included 22 countries, the average value of indicators evaluating the gender inequalities in life expectancy equaled 0.7334 and the average value of indicators evaluating the prosperity of economies equaled 0.3147. Both these values were satisfactory. Luxembourg was definitely closer to the first cluster than to the second. The countries of the first cluster were evaluated positively in terms of the analyzed indicators. The second cluster consisted of 13 countries and these countries took poor outcomes in terms of the analyzed indicators. The average value of indicators evaluating the gender inequalities in life expectancy equaled 0.3932 and the average value of indicators evaluating the prosperity of economies equaled 0.3141. The last third cluster included only Luxembourg that had the average value of gender inequalities in life expectancy (LE_eval) of 0.5246 and the average economic prosperity (GPPpc_eval) of 1.

**Figure 2:**
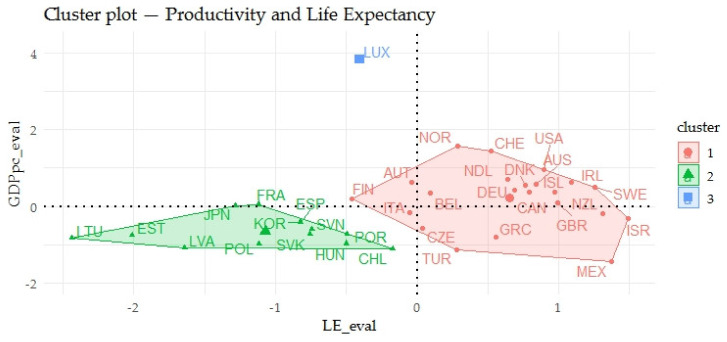
Clusters of OECD countries—the relations of economic prosperity and gender inequalities in life expectancy.

The previous Figure 2 shows the relations of indicators evaluating the gender inequalities in life expectancy (LE_eval) and indicators evaluating the prosperity of economies (GPPpc_eval) in two-dimensional space. In the above output, a certain relationship could be seen. The relationship between LE_eval and GPPpc_eval was evaluated using a nonparametric test of Spearman’s ρ, which at the *p*-value of 0.0143 took the value of the correlation coefficient of 0.4049, interpreted as a medium to substantial rate of association. Focusing on the individual countries, it could be seen that the Baltic countries such as Lithuania, Latvia and Estonia showed very negative outputs of the evaluation and Norway, Switzerland or the USA showed very positive outputs.

**Figure 3:**
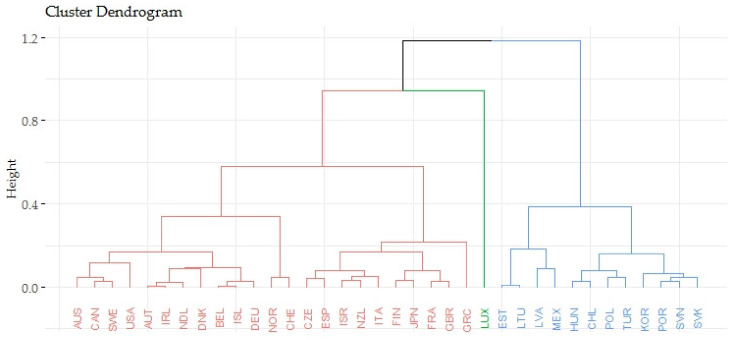
Hierarchical clusters of OECD countries—the relations of economic prosperity and gender inequalities in causes of mortality.

Based on the agglomerative coefficient of approximately 0.9416, the Ward’s method was considered the most appropriate method. The Silhouette method recommended three clusters for the application. The following Figure 3 shows the output of the dendrogram, which determines the links of indicators evaluating the gender inequalities in the causes of mortality (CM_eval) and indicators evaluating the economic prosperity of countries (GDPpc_eval).

The previous Figure 3 focuses on the relations of indicators evaluating the gender inequalities in the causes of mortality (CM_eval) and indicators evaluating the prosperity of economies (GPPpc_eval). The countries were divided into three groups and the order of these clusters was very similar to the previous case. In the first cluster, there were 23 countries and the average value of indicators evaluating the gender inequalities in the causes of mortality equaled 0.7044. The average value of indicators evaluating the prosperity of economies equaled 0.3311. The second cluster included 12 countries and the average value of indicators evaluating the gender inequalities in the causes of mortality equaled 0.5497. The average value of indicators evaluating the prosperity of economies equaled 0.1038. It could be concluded that the second cluster contained the countries that had the lowest outcomes in terms of the analyzed indicators. The last third cluster included only Luxembourg that had the average value of indicator evaluating the economic prosperity of 1, and the average value of indicator evaluating the gender inequalities in the causes of mortality equaled 0.5735.

**Figure 4:**
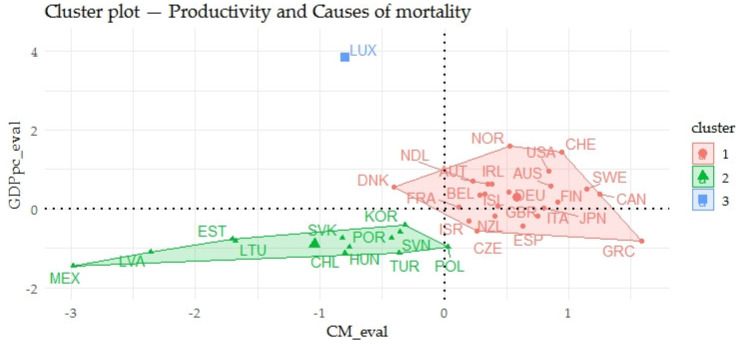
Clusters of OECD—the relations of economic prosperity and gender inequalities in causes of mortality.

The Figure 4 presents the relations of indicators evaluating the gender inequalities in the causes of mortality (CM_eval) and indicators evaluating the prosperity of economies (GPPpc_eval) in two-dimensional space. In the above outputs, a certain relationship could be seen. The relationship between CM_eval and GPPpc_eval was evaluated using a nonparametric test of Spearman’s ρ, which at the *p*-value of 0.0004 took the value of the correlation coefficient of 0.5568, interpreted as a substantial rate of association. Focusing on the individual countries, it could be seen that Mexico and the Baltic countries such as Lithuania, Latvia and Estonia showed very negative outputs of the evaluation and the countries such as Norway, Switzerland or the USA showed very positive outputs.

**Figure 5:**
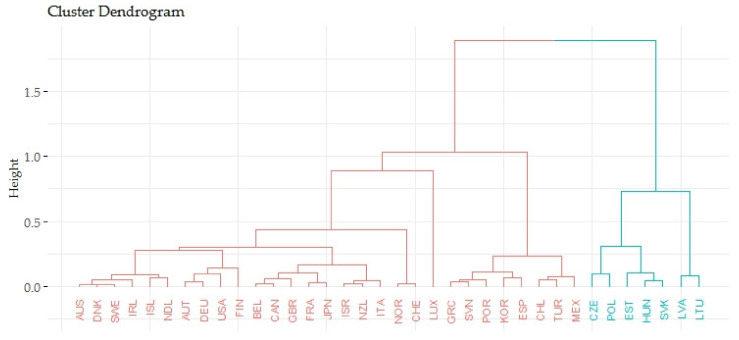
Hierarchical clusters of OECD countries—the relations of economic prosperity and gender inequalities in avoidable mortality.

Based on the agglomerative coefficient of approximately 0.9592, the Ward’s method was considered the most appropriate method. The Silhouette method recommended two clusters for the application. The following Figure 5 shows the output of the dendrogram, which determined the links of indicators evaluating the gender inequalities in avoidable mortality (AM_eval) and indicators evaluating the economic prosperity of countries (GPPpc_eval).

The previous Figure 5 focuses on the relations of indicators evaluating the gender inequalities in avoidable mortality (AM_eval) and indicators evaluating the prosperity of economies (GPPpc_eval). The countries were divided into two groups based on the smallest differences within the cluster and the largest differences between other countries (clusters). The first cluster consisted of 29 countries and the average value of indicators evaluating the gender inequalities in avoidable mortality equaled 0.8675. The average value of indicators evaluating the prosperity of economies equaled 0.3125. The countries of the first cluster were evaluated positively in terms of the analyzed indicators. The second cluster included seven countries and these countries had the lowest outcomes in terms of the analyzed indicators. The average value of indicators evaluating the gender inequalities in avoidable mortality equaled 0.3413 and the average value of indicators evaluating the prosperity of economies equaled 0.114.

**Figure 6:**
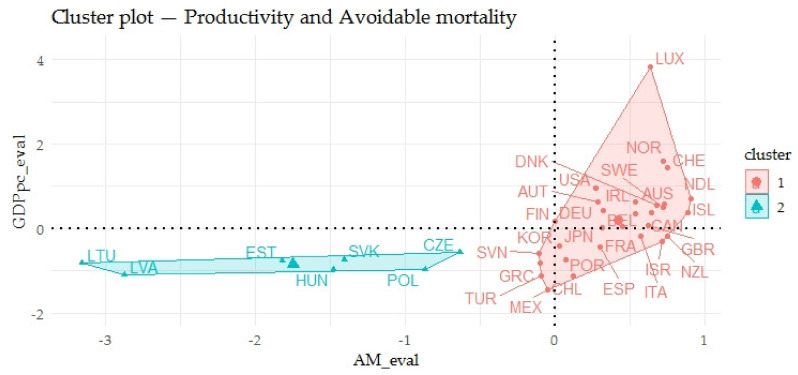
Clusters of OECD—the relations of economic prosperity and gender inequalities in avoidable mortality.

The previous Figure 6 shows the relations of indicators evaluating the gender inequalities in avoidable mortality (AM_eval) and indicators evaluating the prosperity of economies (GPPpc_eval) in two-dimensional space. In the above output, a certain relationship could be seen. The relationship between AM_eval and GPPpc_eval was evaluated using a nonparametric test of Spearman’s ρ, which at the *p*-value of 1.5266E-7 took the value of the correlation coefficient of 0.7485, interpreted as a substantial to strong rate of association. Focusing on the individual countries, it can be seen that the Baltic countries such as Lithuania, Latvia and Estonia showed very negative outputs of the evaluation and the countries such as Luxembourg, Norway, Switzerland or the USA showed very positive outputs.

## 5. Discussion

Health of the population is a very valuable economic commodity of each country. For this reason, the health is an important source of comparative economic [7]. Therefore, it is not surprising that many authors emphasized the importance of health in the economic dimension [15,18,19,21,79]. On this basis, it can be considered that the efforts leading to better population health contribute to creating richer economies [20]. The fact is that the population is aging, a very interesting idea is offered by Dziuba et al. [80], who examined the use of modern digital technologies by the older generation, which can favorably prolong their life activities and make everyday life easier. Developed economies are interested in achieving the health potential of all groups of the population and therefore the policy makers should be focused on the inequalities in health outcomes. The health inequality is also an important element for assessing the countries [9,29]. The gender inequalities in health have been examined in several studies and the results revealed the differences between women and men in many health indicators reflecting morbidity or mortality [41,42]. The present study highlights the health in economic dimension and evaluates the relations between gender inequalities in health and the prosperity of economies.

In the first step of the analytical process, a descriptive analysis, a difference analysis and a relationship analysis were used. The output of descriptive analysis revealed that the most frequent causes of mortality were neoplasms (CM_2) and diseases of the circulatory system (CM_7). As evidenced by the research findings in many other studies [38,39,40], this study also confirmed the gender inequalities in health. In the vast majority of analyzed health indicators, including a life expectancy, the causes of mortality and an avoidable mortality, there was a significant difference between men and women. In most of the indicators, more positive health outcomes were identified in women, thus it could be assumed that women were healthier in terms of selected indicators. A significant disadvantage of men was evident in mortality due to neoplasms (CM_2), while Ellison [56] emphasized that male mortality is higher in 13 of the 18 types of cancer. The results showed that women lived longer and men died earlier and this is in accordance with the findings of several international studies [51,61,62,65] and the studies focused on the specific areas of causes of mortality [52,53,54,55]. In terms of the avoidable mortality, the results are in line with the findings of other studies by Lefevre et al. [49] and de Abreu et al. [50]. The only health indicator of more positive results in men was CM_11, diseases of the musculoskeletal system and connective tissue, and this result agreed with the fact that women have a disadvantage in musculoskeletal pain and mobility [43,44,45]. From the point of view of the relationship analysis, it could be concluded that there was a significant relationship between the vast majority of health indicators and the economic prosperity represented by GDP.

In the next step of the analysis, a multiple regression analysis was used to assess the effect of selected health indicators (LE, CM and AM) on the prosperity of economies. Thus, three separate regression models were created. Each model was specific to one group of health indicators used as independent variables. The effect of male and female health indicators was evident, in general, it could be confirmed that the better the health indicators, the higher economic prosperity was expected and vice versa. These results may be supported by other claims that poor health outcomes are reflected in economic losses [24,81] and better health outcomes bring economic benefits [22,23]. Other results revealed a significant effect of gender inequalities in life expectancy at birth (LE_1) on the economic prosperity represented by GDP. A negative coefficient indicates that if the gender inequality is decreased in LE_1, the economic prosperity is expected to increase. It can be discussed whether it is more effective to reduce the inequalities on the part of women or men. The effect of life expectancy at birth on the economic prosperity was significant for both men and women, but the effect of men was statistically more significant. Based on this, it could be assumed that men are more productive, and therefore the efforts to increase life expectancy at birth of men would be more effective in terms of the economic prosperity. Regarding the gender inequalities in the causes of mortality, a significant effect on the economic prosperity was confirmed for two indicators, i.e., neoplasms (CM_2) and diseases of the circulatory system (CM_7). Both variables had a negative coefficient, thus if the gender inequality in these indicators decreases, an increase of the economic prosperity is expected. Based on the significance of the male and female coefficients, the results showed that the initiatives aimed at reducing the gender inequalities in mortality from neoplasms (CM_2) seem to be more effective on the part of men than women. In terms of gender inequalities in avoidable mortality, a significant effect on the economic prosperity was confirmed in preventable mortality (AM_1). Based on a negative coefficient, it could be concluded that if the gender inequality is decreased in AM_1, the economic prosperity is expected to increase. When assessing the significance of the effects of men and women, it could be considered that efforts to reduce the gender inequalities in preventable mortality should focus on men who showed a more significant coefficient. These findings confirmed the importance of efforts to close the gender gap in health in order to achieve economic gains. Similar results were found in the socioeconomic, racial and ethnic dimensions [11,12,71,72].

In the last part of the regression analysis, a simple regression model was used, in which the indicator of economic prosperity was used as a dependent variable and all health indicators in the specification of men, women and gender inequalities were used as independent variables. The univariate view of the effect of selected health indicators on the economic prosperity revealed that most health indicators in the specification of gender inequalities showed a significant effect on the prosperity of economies. The most significant effects were found in variables: life expectancy at birth (LE_1), neoplasms (CM_2), diseases of the circulatory system (CM_7), preventable mortality (AM_1) and treatable mortality (AM_2). All these health variables had a negative β coefficient. Thus, if the gender inequality in health decreases, the economic prosperity is expected to increase. In terms of the prosperity of economies, the efforts to reduce the negative values of these health indicators seem to be more effective on the part of men than women.

The last part of the analyses included the assessment of countries in terms of the links of the indicators evaluating the gender inequalities in health and indicators evaluating the economic prosperity of countries. Focusing on the indicators evaluating the gender inequalities in life expectancy (LE_eval) and indicators evaluating the prosperity of economies (GDPpc_eval), the OECD countries were divided into three clusters. From the point of view of these indicators, the Baltic countries such as Lithuania, Latvia and Estonia showed very negative outputs of the evaluation and Norway, Switzerland or the USA showed very positive outputs. With a focus on the links of the indicators evaluating the gender inequalities in the causes of mortality (CM_eval) and indicators evaluating the prosperity of economies (GDPpc_eval), the OECD countries were divided into three clusters. It can be concluded that Mexico and Latvia showed very negative outputs of the evaluation and the countries such as Norway, Switzerland or the USA showed very positive outputs. In the assessment of the relations of the indicators evaluating the gender inequalities in avoidable mortality (AM_eval) and those evaluating the prosperity of economies (GPPpc_eval), the OECD countries were divided into two clusters. In this evaluation, it can also be stated that Lithuania, Latvia and Estonia showed very negative outputs and the countries such as Luxembourg, Norway, Switzerland or the USA showed very positive outputs.

The application of cluster analysis pointed to individual countries that were identified in positive or negative optics in terms of the indicators evaluating the gender inequalities in health and those evaluating the prosperity of economies. It should be noted that most countries were identified in positive optics. The increased attention should be paid to countries such as Lithuania, Latvia, Estonia, Poland, Hungary or Slovakia in reducing regional disparities in this issue.

Based on the above-mentioned findings, it can be concluded the presence of gender inequalities in health between the selected developed countries. These findings support the recommendations of the OECD and the WHO, which urge the policy makers to address this serious issue in the field of health. At this point, it can be underlined that health inequalities are the result of many social, economic and environmental factors [9,29,30,32,36]. Therefore, the public policies should address this global health problem from different perspectives and use all available tools to reduce these inequalities. These tools can be effective financing of health, health accessibility, quality health care, social protection, education support, as well as increasing the well-being of the population. All these tools should be focused on the population as a whole, but disadvantaged groups are at the greatest health risk. In general, more developed countries are supposed to have effective health care systems that are able to achieve the equity in health, but there are clear differences between population groups. Health inequalities can be considered as a defect in the health systems and it encourages the idea that the gender inequalities in health are an issue to be examined. The importance of this topic is highlighted by the findings of this study that confirmed the effect of gender inequalities on the economic prosperity. One of the main aims of public policies is to increase the economic prosperity of countries, therefore the policy makers should focus on the reduction of gender inequalities in health. The findings of the present research gave to the decision makers an additional information source to achieve economic gains. From this point of view, it could be recommended the reduction of gender inequalities on the part of men in terms of an effective increase of economic prosperity. These political implications represent a significant added value of the present study. In general, it can be assumed that reducing health inequalities between different groups of the population can bring various economic benefits. On this basis, it can be stated that the issue of health inequalities seems to be overlooked in countries, and the policy makers are not aware of the importance of health inequalities in terms of their economic effects. This study shows that the policy efforts to reduce health inequalities are beneficial not only in the life of the individual but also in the economic life. This could be a driving force for improving the health systems and improving the distribution of health care in developed countries, with the most pronounced effect expected for disadvantaged groups.

## 6. Conclusions

As the evidence shows, the health of the population is a representative element of the country and at the same time health has a significant effect on the prosperity of economies. Therefore, it is necessary to emphasize the health and economic outcomes of individual countries. The health inequalities are the results of the specificity of countries, their geographical location, social or economic aspects. On the other hand, the strategic policies of countries have a major impact on reducing or deepening the inequalities. Based on this, the policy makers should carefully assess the indicators of health and the inequalities in health, while these indicators should definitely not be missing in assessing the economic prosperity of countries. For this reason, this study is focused on analyzing the gender inequality in health and the economic prosperity represented by GDP.

The primary aim of the research in this study was to evaluate the relations between gender health inequalities and the economic prosperity of OECD countries. This purpose was decomposed into several research questions that are mentioned in the methodological part of this study. Based on the results of several analytical processes, the relationship between the health of the population and the prosperity of economies can be confirmed. The main purpose of the research was to prove the effect of selected health indicators (in the specification of men, women and gender inequalities) on the economic prosperity represented by GDP. In several cases, the significant effects were confirmed and it could be concluded that the main objective was met. Based on the research findings, it could be emphasized that the health policies should be more focused on reducing gender health inequalities on the part of men, in order to effectively increase the economic prosperity represented by the GDP. In general, it was also concluded that the effect of specific areas of causes of mortality could be explained by their frequency. As the indicators of avoidable mortality fundamentally affected the prosperity of economies, the policy makers should be interested in this issue and should take the necessary activities to reduce the avoidable mortality. Despite the fact that the reduction of regional health disparities is a proclaimed issue in both scientific and applied discussions, it can be stated that the significant gender differences were identified between OECD countries. Therefore, the health policies should be more proactive in order to reduce this gender inequality in health.

A potential limitation of this study might be a fact that the collected data represent the economically developed countries (OECD) and the findings could be generalized only to similar countries. Due to the high frequency of missing data since 2017, the comprehensive time series offered by the database did not enter the analysis. The lower bound was chosen with the respect to the effect of time; the larger the time series, the higher the likelihood of hidden effects. The sample size is considered adequate to support the formulated conclusions.

This study mapped the health areas, while future research will focus on analyzing the specific diagnoses and their effect on economic prosperity in terms of socioeconomic inequality. Future research will also focus on comparing countries with different level of economic development. Last but not least, future research will reveal the effect of health indicators on economic parameters other than the prosperity of countries.

## Figures and Tables

**Table 1:** Descriptive analysis results.

Gender	Stat. Char.	Life Expectancy					Causes of Mortality													Avoidable Mortality	
		LE_1	LE_2	LE_3	LE_4	LE_5	CM_1	CM_2	CM_3	CM_4	CM_5	CM_6	CM_7	CM_8	CM_9	CM_10	CM_11	CM_12	CM_13	AM_1	AM_2
F	missing	0	0	0	0	0	12	12	13	12	13	12	12	12	12	14	12	12	13	17	17
	Mean	82.86	43.78	25.29	21.02	9.74	11.43	165.76	2.33	26.64	24.39	28.54	251.32	50.37	27.42	1.65	4.56	13.80	2.35	78.31	67.05
	CI 95 L	82.58	43.53	25.08	20.83	9.63	10.68	162.33	2.16	22.97	22.17	26.42	236.41	47.72	26.10	1.48	4.33	12.97	2.18	74.83	63.89
	CI 95 U	83.14	44.03	25.50	21.21	9.85	12.18	169.19	2.50	30.31	26.62	30.65	266.24	53.02	28.74	1.83	4.79	14.63	2.52	81.79	70.21
	SD.	2.18	1.95	1.62	1.47	0.86	5.80	26.51	1.31	28.38	17.20	16.36	115.33	20.49	10.20	1.35	1.80	6.43	1.32	26.89	24.42
	Skew	−0.82	−0.69	−0.54	−0.52	−0.34	1.50	−0.28	1.91	4.32	0.33	2.39	1.29	0.22	2.19	2.59	0.48	1.07	1.95	1.10	1.38
	Kurt	0.34	0.21	0.54	0.81	1.48	3.99	−0.05	5.23	20.08	−0.84	8.53	0.61	−0.51	6.07	8.29	0.24	0.81	4.61	0.62	1.26
M	missing	0	0	0	0	0	12	12	13	12	13	12	12	12	12	16	12	12	13	17	17
	Mean	77.21	38.79	21.34	17.56	7.86	16.99	280.26	2.67	32.99	25.53	33.83	364.83	89.68	45.63	1.59	3.42	20.01	2.91	210.92	94.67
	CI 95 L	76.77	38.41	21.08	17.35	7.75	15.94	273.30	2.49	29.07	23.65	31.73	340.90	86.16	42.79	1.41	3.25	18.88	2.70	198.03	88.07
	CI 95 U	77.65	39.17	21.60	17.78	7.97	18.05	287.22	2.86	36.92	27.41	35.94	388.77	93.19	48.46	1.78	3.60	21.14	3.13	223.82	101.27
	SD.	3.40	2.90	2.01	1.65	0.85	8.14	53.55	1.44	30.20	14.46	16.21	184.21	27.04	21.80	1.40	1.33	8.69	1.66	99.26	50.78
	Skew	−1.15	−1.17	−1.03	−0.96	−0.60	0.92	0.28	1.71	4.20	0.19	2.62	1.58	0.39	1.69	3.20	0.19	1.37	2.10	1.69	1.57
	Kurt	0.30	0.44	0.00	−0.10	−0.25	1.41	0.51	4.38	19.32	−0.77	10.16	1.55	−0.71	2.82	12.40	−0.25	1.69	5.36	2.22	1.63
Dif. Test	SW F	0.94 †	0.95 †	0.96 †	0.96 †	0.95 †	0.89 †	0.98 †	0.84 †	0.49 †	0.95 **	0.79 †	0.83 †	0.98 †	0.79 †	0.74 †	0.98 *	0.92 †	0.81 †	0.89 †	0.85 †
	SW M	0.86 †	0.86 †	0.88 †	0.89 †	0.96 †	0.94 †	0.95 **	0.87 †	0.52 †	0.98 †	0.77 †	0.78 †	0.96 **	0.82 †	0.67 †	0.99 **	0.87 †	0.79 †	0.79 †	0.78 †
	W	59527 †	59594 †	60036 †	60112 †	59760 †	16590 †	1428.5 †	23363 †	20744 †	26855 •	20970 †	14277 †	38864 †	9726.5 †	29016 •	39187 †	15014 †	20426 †	1239.5 †	16393 †

Note. F—Female; M—Male; missing—number of missing observations, Mean—arithmetic mean; CI 95 L—95% confidence interval lower bound; CI 95 B—95% confidence interval upper bound, SD—standard deviation; Skew—skewness, Kurt—kurtosis, SW—Shapiro–Wilk Normality Test value, W—Wilcoxon Test value. •—*p* value < 0.1; *—*p* value < 0.05; **—*p* value < 0.01; †—*p* value < 0.001

**Table 2:** Descriptive statistics of gender inequalities in health indicators.

**Descriptive characteristics**	**LE_1**	**LE_2**	**LE_3**	**LE_4**	**LE_5**
Mean	5.64	4.99	3.94	3.45	1.90
CI 95 L	5.40	4.77	3.79	3.33	1.77
CI 95 U	5.88	5.21	4.08	3.57	2.03
SD.	1.85	1.67	1.12	0.91	1.00
**Descriptive characteristics**	**CM_1**	**CM_2**	**CM_3**	**CM_4**	**CM_5**
Mean	5.65	114.62	0.49	6.49	4.39
CI 95 L	5.15	108.70	0.44	6.02	3.98
CI 95 U	6.14	120.55	0.54	6.95	4.80
SD.	3.78	45.51	0.38	3.57	3.15
**Descriptive characteristics**	**CM_6**	**CM_7**	**CM_8**	**CM_9**	**CM_10**
Mean	5.36	113.00	39.57	18.28	0.31
CI 95 L	5.04	103.47	37.39	16.61	0.27
CI 95 U	5.68	122.53	41.76	19.96	0.35
SD.	2.45	73.18	16.78	12.87	0.30
**Descriptive characteristics**	**CM_11**	**CM_12**	**CM_13**	**AM_1**	**AM_2**
Mean	1.25	6.18	0.62	132.83	27.82
CI 95 L	1.12	5.76	0.56	122.65	23.89
CI 95 U	1.37	6.60	0.68	143.02	31.75
SD.	0.95	3.20	0.45	78.21	30.17

**Table 3:** Correlation analysis output.

**Variable**	**LE_1**	**LE_2**	**LE_3**	**LE_4**	**LE_5**
Female	0.4407 †	0.4118 †	0.4248 †	0.4436 †	0.4614 †
Male	0.5942 †	0.5708 †	0.5404 †	0.5422 †	−0.0833
Inequal	−0.5879 †	−0.4686 †	−0.3642 †	−0.3257 †	0.4347 †
**Variable**	**CM_1**	**CM_2**	**CM_3**	**CM_4**	**CM_5**
Female	0.0985	0.0997	0.1850 **	−0.2614 †	0.6888 †
Male	−0.1129 •	−0.2969 †	0.1804	−0.1885 **	0.6948 †
Inequal	−0.3016 †	−0.4318 †	0.0914	0.0342	0.0999
**Variable**	**CM_6**	**CM_7**	**CM_8**	**CM_9**	**CM_10**
Female	0.6079 †	−0.4228 †	0.072	−0.2871 †	−0.0386
Male	0.6339 †	−0.3708 †	−0.2222 †	−0.4879 †	−0.0845
Inequal	−0.002	−0.1379 *	−0.4873 †	−0.5780 †	−0.0966
**Variable**	**CM_11**	**CM_12**	**CM_13**	**AM_1**	**AM_2**
Female	0.2094 *	−0.1193 •	−0.2979 †	−0.3431 †	−0.5384 †
Male	0.1705 **	−0.1133 •	−0.3172 †	−0.6030 †	−0.6787 †
Inequal	0.0971	−0.1833 **	−0.1793 **	−0.6741 †	−0.7056 †

Note: •—*p* value < 0.1; *—*p* value < 0.05; **—*p* value < 0.01; †—*p* value < 0.001.

**Table 4:** Assumptions for the regression model.

Statistic	LE -> GDPpc			CM -> GDPpc			AM -> GDPpc		
	Model 1 F	Model 1 M	Model 1 Inq	Model 2 F	Model 2 M	Model 2 Inq	Model 3 F	Model 3 M	Model 3 Inq
VIF	LE _2 - LE_4	LE _2 - LE_4	LE _2, LE_3	CM_4, CM_12	CM_4, CM_13	-	-	AM_1	AM_2
F Test country	70.18 †	141.19 †	102.84 †	126.29 †	101.14 †	102.35 †	133.46 †	145.89 †	133.53 †
Hausman	7.15 *	5.62 •	7.45 •	1.96	34.80 †	12.65	18.71 †	3.12 •	1.6
Breusch Pagan	41.18 †	51.83 †	70.18 †	195.06 †	198.43 †	252.66 †	23.28 †	14.90 †	11.1 †
Regression model	plm - fixed effect; Arellano	plm - random effect; White 1	plm - random effect; White 1	plm - random effect; White 1	plm - fixed effect; Arellano	plm - random effect; White 1	plm - fixed effect; Arellano	plm - random effect; White 1	plm - random effect; White 1

Note: •—*p* value < 0.1; *—*p* value < 0.05; **—*p* value < 0.01; †—*p* value < 0.001.

**Table 5:** Regression analysis output—a multivariate approach to the relations of life expectancy and economic prosperity.

LE->GDPpc	Model 1 F (R2 = 0.40)		Model 1 M (R2 = 0.42)		Model 1 Ing (R2 = 0.27)	
	Estimate	Pr(>|t|)	Estimate	Pr(>|t|)	Estimate	Pr(>|t|)
Intercept	-	-	−233,574.28	<2.2 × 10^−16^	64,125.03	<2.2 × 10^−16^
LE_1	8025.8	6.6 × 10^−8^	3627.69	<2.2 × 10^−16^	−6259.97	4.0 × 10^−12^
LE_4	-	-	-	-	1638.92	2.8 × 10^−1^
LE_5	−7503.7	6.9 × 10^−5^	−1006.97	1.5 × 10^−1^	2184.58	3.7 × 10^−2^

**Table 6:** Regression analysis output—a multivariate approach to the relations of mortality causes and economic prosperity.

CM->GDPpc	Model 2 F (R2 = 0.48)		Model 2 M (R2 = 0.54)		Model 2 Inq (R2 = 0.36)	
	Estimate	Pr(>|t|)	Estimate	Pr(>|t|)	Estimate	Pr(>|t|)
Intercept	63,687.09	< 2.2 × 10^−16^	-	-	58,174.04	< 2.2 × 10^−16^
CM_1	−144.211	8.6 × 10^−2^	36.97	5.7 × 10^−1^	−12.515	9.4 × 10^−1^
CM_2	−122.316	2.5 × 10^−3^	−96.39	1.8 × 10^−5^	−90.327	1.8 × 10^−3^
CM_3	−460.226	3.8 × 10^−1^	456.72	2.54 × 10^−1^	864.435	1.8 × 10^−1^
CM_4	-	-	-	-	155.58	1.9 × 10^−1^
CM_5	230.205	2.9 × 10^−8^	176.59	2.0 × 10^−5^	131.632	2.6 × 10^−1^
CM_6	102.722	2.4 × 10^−2^	93.23	4.8 × 10^−2^	228.777	7.1 × 10^−2^
CM_7	−36.425	4.9 × 10^−4^	−28.56	7.4 × 10^−4^	−54.804	3.9 × 10^−4^
CM_8	125.42	2.2 × 10^−3^	67.09	1.6 × 10^−2^	−76.498	1.5 × 10^−1^
CM_9	−288.121	2.0 × 10^−3^	−77.73	1.7 × 10^−1^	−96.719	2.2 × 10^−1^
CM_10	717.806	7.8 × 10^−2^	727.37	6.8 × 10^−2^	1090.157	2.1 × 10^−1^
CM_11	−316.012	3.2 × 10^−1^	91.85	7.8 × 10^−1^	−413.966	3.1 × 10^−1^
CM_12	-	-	-	-	−293.186	5.0 × 10^−2^
CM_13	198.791	6.3 × 10^−1^	246.31	5.2 × 10^−1^	735.951	2.2 × 10^−1^

**Table 7:** Regression analysis output—a multivariate approach to the relations of avoidable mortality and economic prosperity.

LE->GDPpc	Model 3 F (R2 = 0.35)		Model 3 M (R2 = 0.31)		Model 3 Inq (R2 = 0.27)	
	Estimate	Pr(>|t|)	Estimate	Pr(>|t|)	Estimate	Pr(>|t|)
Intercept	-	-	62,562.81	< 2.2 × 10^−16^	56,429.77	< 2.2 × 10^−16^
AM_1	−284.726	1.1 × 10^−3^	−113.70	< 2.2 × 10^−16^	−134.46	< 2.2 × 10^−16^
AM_2	−234.304	4.4 × 10^−2^	-	-	-	-

**Table 8:** Regression analysis output—a univariate model.

Model	Female				Male				Gender Inequalities			
	R2	α	β	Pr(>|t|)	R2	α	β	Pr(>|t|)	R2	α	β	Pr(>|t|)
**LE_1**	0.30	−273,771.7 †	3771.8 d	7.1 × 10^−15^	0.41	−225,031.7 †	3414.9 d	<2.2 × 10^−16^	0.22	69,254.5†	−5428.2 d	5.7 × 10^−10^
**LE_2**	0.25	−112,231.9 †	3449.1 d	8.9 × 10^−12^	0.44	-	4278.1 c	3.2 × 10^−10^	0.13	59,785.3†	−4229.3 d	9.1 × 10^−5^
**LE_3**	0.22	−54,449.2 †	36,878.0 d	1.8 × 10^−11^	0.40	−70,894.0 †	5133.7 d	<2.2 × 10^−16^	0.07	53,106.8†	−3655.4 d	9.2 × 10^−3^
**LE_4**	0.21	−41,592.9 †	3825.9 d	7.6 × 10^−16^	0.37	−54,014.1 †	5278.2 d	<2.2 × 10^−16^	0.02	50,001.1†	−3270.3 d	1.9 × 10^−2^
**LE_5**	0.15	−4880.4 †	4491.8 d	2.6 × 10^−11^	0.09	-	3696.0 c	3.9 × 10^−2^	0.02	-	1970.9 c	5.1 × 10^−2^
**CM_1**	0.00	37,645.9 †	83.9 b	4.2 × 10^−1^	0.00	38,181.2 †	24.0 d	8.3 × 10^−1^	0.01	40,404.3†	−336.8 d	1.8 × 10^−1^
**CM_2**	0.18	-	−300.5 b	3.3 × 10^−10^	0.31	85,473.2 †	−166.8 d	2.4 × 10^−13^	0.26	61,033.4†	−195.5 d	7.5 × 10^−15^
**CM_3**	0.01	41,223.5 †	−1142.2 d	8.4 × 10^−2^	0.01	36,350.1 †	845.4 d	2.5 × 10^−1^	0.03	-	1781.5 c	1.2 × 10^−1^
**CM_4**	0.01	41,788.2 †	−124.7 d	1.2 × 10^−2^	0.00	40,196.6 †	−50.7 d	3.1 × 10^−1^	0.01	37,430.4†	178.8 d	2.8 × 10^−1^
**CM_5**	0.26	29,488.2 †	379.7 d	4.1 × 10^−10^	0.29	28,259.5 †	408.9 d	5.8 × 10^−10^	0.02	37,155.7†	332.2 b	1.7 × 10^−2^
**CM_6**	0.15	29,501.7 †	315.9 b	5.2 × 10^−11^	0.18	26,930.67 †	343.7 d	5.1 × 10^−10^	0.01	37,073.3†	278.9 d	1.4 × 10^−1^
**CM_7**	0.23	57,544.8 †	−74.4 d	5.0 × 10^−11^	0.28	59,268.0 †	−56.2 d	7.4 × 10^−15^	0.17	49,032.5†	−92.2 d	1.2 × 10^−12^
**CM_8**	0.00	36,562.8 †	39.8 b	3.8 × 10^−1^	0.01	44,292.0 †	−64.2 d	4.2 × 10^−2^	0.10	49,216.3†	−274.9 d	3.6 × 10^−7^
**CM_9**	0.14	54,157.6 †	−572.2 d	1.3 × 10^−6^	0.14	53,775.3 †	−335.4 d	6.1 × 10^−8^	0.04	-	−279.5 c	1.1 × 10^−3^
**CM_10**	0.00	38,372.5 †	150.9 d	6.8 × 10^−1^	0.02	38,247.1 †	195.7 d	6.4 × 10^−1^	0.00	-	621.6 c	6.4 × 10^−1^
**CM_11**	0.00	39,521.1 †	−210.0 b	5.4 × 10^−1^	0.00	38,994.9 †	−123.6 d	8.1 × 10^−1^	0.00	38,887.7†	−252.4 d	6.3 × 10^−1^
**CM_12**	0.00	40,564.0 †	−146.1 d	2.4 × 10^−1^	0.05	44,737.5 †	−313.8 b	4.9 × 10^−4^	0.08	42,441.3†	−640.5 d	2.9 × 10^−6^
**CM_13**	0.00	-	−245.5 c	7.9 × 10^−1^	0.00	-	−372.4 c	6.4 × 10^−1^	0.00	38,122.8†	678.8 d	3.2 × 10^−1^
**AM_1**	0.31	-	−445.2 c	2.5 × 10^−15^	0.31	62,562.8 †	−113.7 d	<2.2 × 10^−16^	0.27	56,429.8†	−134.5 d	<2.2×10^−16^
**AM_2**	0.28	65,977.1 †	−407.4 b	<2.2×10^−16^	0.29	60,766.5 †	−233.2 d	<2.2 × 10^−16^	0.16	47,238.5†	−308.0 d	5.0 × 10^−11^

Note: •—*p* value < 0.1; *—*p* value < 0.05; **—*p* value < 0.01; †—*p* value < 0.001. Note 2: b—fixed, c—random white 1, d—fixed arrelano.
